# Implications of expansin-like 3 gene in *Dictyostelium* morphogenesis

**DOI:** 10.1186/s40064-015-0964-0

**Published:** 2015-04-19

**Authors:** Takefumi Kawata, Yuri Nakamura, Yukika Saga, Yumi Iwade, Megumi Ishikawa, Aya Sakurai, Nao Shimada

**Affiliations:** Department of Biology, Faculty of Science, Toho University, 2-2-1 Miyama, Funabashi, Chiba 274-8510 Japan; Present Address: Department of Basic Science, Graduate School of Arts and Sciences, University of Tokyo, 3-8-1 Komaba, Meguro-ku, Tokyo 153-8902 Japan

**Keywords:** Cell wall, Cellulose-binding, *Dictyostelium*, Expansin, Morphogenesis, STAT transcription factor

## Abstract

*Dictyostelium* harbors multiple *expansin-like* genes with generally unknown functions. Thus, we analyzed the *expansin-like 3* (*expL3*) gene and found that its expression was reduced in a null mutant for a *STATa* gene encoding a transcription factor. The expression of *expL3* was developmentally regulated and its transcript was spliced only in the multicellular stages. The *expL3* promoter was activated in the anterior prestalk region of the parental strain and downregulated in the *STATa* null slug, although the *expL3* promoter was still expressed in the prestalk region. The *expL3* overexpressing strain exhibited delayed development and occasionally formed an aberrant structure, i.e., a fruiting body-like structure with a short stalk. The ExpL3-myc protein bound cellulose.

**Electronic supplementary material:**

The online version of this article (doi:10.1186/s40064-015-0964-0) contains supplementary material, which is available to authorized users.

## Background

Expansins are cell wall proteins in plants that loosen the cell wall, thereby regulating cell wall enlargement in growing cells. Expansins may act by breaking the noncovalent bonds among wall polysaccharides (Cosgrove 2005). Expansin activity is also associated with morphogenesis and other developmental events, such as leaf primordium formation, fruit ripening, xylem formation, pollination, seed germination, and abscission (Sampedro and Cosgrove 2005). Plant expansins comprise a large gene superfamily of four divergent families: α-expansin (EXPA), β-expansin (EXPB), expansin-like A (EXLA), and expansin-like B (EXLB) (Sampedro and Cosgrove 2005).

The existence of expansin-like proteins is also known in bacteria and fungi, such as EXLX1 in *Bacillus subtilis*, which is structurally and functionally similar to plant expansins. The cellular slime mold *Dictyostelium* is a lower eukaryote, which produces a cellulose-based cell wall. It is a rare species that harbors multiple expansin-like genes in its genome, i.e., at least nine genes (*expL1–9*) (Darley et al. 2003; Ogasawara et al. 2009), although *expL4* appears to be a putative pseudogene (http://dictybase.org). It is of evolutionary importance to understand the role of expansin-like molecules in *Dictyostelium*, which is significantly divergent from plants.

In a previous study, we demonstrated the role of an expansin-like gene, *expL7*, which regulates morphogenesis during *Dictyostelium* development (Ogasawara et al. 2009). The expression of *expL7* is under the control of CudA, a putative transcription factor (Wang and Williams 2010). STATa is a transcription factor that activates the expression of *cudA* in the anterior prestalk tip region (Fukuzawa and Williams 2000), thus STATa indirectly controls *expL7* gene expression. Slugs of both *STATa* and *cudA* null mutants tend to migrate for longer (slugger phenotype) and they fail to culminate, eventually forming an aberrant structure (Fukuzawa et al*.*^1997^; Mohanty et al. 1999). The anterior prestalk region serves as an organizer during multicellular development in *Dictyostelium*, and the region where STATa is activated to express *cudA* is designated as the “tip-organizer.” In this study, we analyzed the function of another expansin-like gene, *expL3*, which was positively regulated by STATa.

## Materials and methods

### Cells and growth conditions

*Dictyostelium discoideum* Ax2 cells were axenically cultured in HL5 medium at 22°C. Cells of the *STATa* null strain were grown in HL5 supplemented with 10 μg/ml blasticidin S (Kaken Pharmaceutical, Japan). The *expL3* null strain was grown in HL5 supplemented with 36 μg/ml hygromycin B (Wako, Japan). Transformants with the *Neo*^*R*^ cassette construct were selected using HL5 supplemented with 20 μg/ml G418 (geneticin; ICN Biochemicals Inc.).

### Analysis of gene expression using semi-quantitative and quantitative RT-PCR

Ax2 and *STATa* null cells were allowed to develop at 22°C on Omnipore filters (JGWP04700, Millipore), which were placed on non-nutrient agar plates. Total RNA was extracted from Ax2 and *STATa* null strains every 3–4 h. cDNA synthesis and RT-PCR were conducted as described previously (Shimada et al. 2004, 2005) using a pair of primers: expL3-RT-1 and expL3-RT-2 or expL3-G7-i and expL3-G8-i. The quantitative RT-PCR analysis was performed as previously described (Shimada et al. 2010). The primers used for RT-PCR are listed in Additional file 1: Table S1.

### *lacZ* fusion construct and β-galactosidase staining

The promoter fragment of the *expL3* gene (the 5’ end point is located 948 nucleotides upstream from the putative translation initiation site) was amplified by PCR to add an *Xba*I site at the 5’ end and a *Bgl*II site at the 3’ end. After digestion with *Xba*I and *Bgl*II, the fragment was gel-purified and subcloned into *Xba*I/*Bgl*II-cut pDdgal-17(H+) (Harwood and Drury 1990) to yield pDdNeo^R^[*expL3/lacZ*]. To detect the promoter activity, cells transformed with pDdNeo^R^[*expL3/lacZ*] were grown and developed on Omnipore filters. Fixation and staining were performed as previously described (Shimada et al. 2005).

### *expL3* expression vectors

The fragment corresponding to the entire open reading frame (ORF) of the *expL3* gene was amplified by PCR using a plasmid DNA containing the *expL3* gene to add a *Sal*I site at the 5’ end and a *Bam*HI site at the 3’ end, before subcloning into pTOPO-Blunt II (Invitrogen) to produce pTOPO[*expL3*-ORF(G4/G5)]. After digestion with *Sal*I and *Bam*HI, the ORF fragment was gel-purified and subcloned into *Sal*I and *Bam*HI-digested pLD1ΔBX-myc (unpublished) to yield pLD1ΔBX[*act15/expL3-*myc]. Plasmid DNA that contained the promoter region of the *ecmF* gene (Shimada et al. 2004) was purified by gel electrophoresis after digesting pLD1ΔBX[*ecmF/dutA*(nF)] (unpublished) with *Sal*I and *Not*I. The *expL3-*myc fragment was purified by gel electrophoresis after digesting pLD1ΔBX[*act15/expL3-*myc] with *Sal*I and *Not*I. Both of the purified DNA fragments were ligated to yield pLD1ΔBX[*ecmF/expL3-*myc]. It should be noted that each of the ExpL3 expression constructs used in this study contained an intron.

### Western blot analysis of ExpL3-myc protein

Cells transformed with pLD1ΔBX[*act15/expL3-*myc] or pLD1ΔBX[*ecmF/expL3-*myc] were allowed to develop until the slug stage, before the slugs were solubilized and analyzed on 7.5% (w/v) SDS-polyacrylamide gels, followed by blotting onto Hybond-C extra filters (Amersham Biosciences, UK). The filters were blocked and detected using the Promega Proto Blot II AP System with Stabilized Substrate, according to the manufacturer’s protocol (Promega). Anti-c-Myc monoclonal antibody 9E10 (1:2000 dilution; Wako) was used as a primary antibody and alkaline phosphatase (AP)-conjugated anti-mouse IgG (1:20,000 dilution; Promega) was used as a secondary antibody.

### Cellulose-binding assay

The cellulose-binding ability of the ExpL3-myc fusion protein was tested according to a previously described procedure (Kunii et al. 2014) with some modifications. First, 2 × 10^7^ cells that overexpressed the ExpL3-myc protein (*act15:expL3 myc*^*OE*^ strain) were allowed to develop until the slug stage on the Omnipore filter (Millipore), before the slugs were harvested and ground with a plastic pestle in the presence of 250 μl of 1 × phosphate-buffered saline (PBS: 137 mM NaCl, 2.7 mM KCl, 8.1 mM Na_2_HPO_4_, 1.47 mM KH_2_PO_4_, pH 7.4) containing cOmplete, Mini, EDTA-free (Roche) as protease inhibitors. Furthermore, 250 μl of 2 × lysis buffer [50 mM KCl, 10 mM Tris–HCl, 2.5 mM MgCl_2_, 0.45% (w/v) Tween 20, pH 8.0] was added to lyse the slug. Microcrystalline cellulose beads (Avicel PH-101; Sigma) were suspended in binding buffer (100 mM Tris–HCl, pH 8.0) at a final concentration of 5% (w/v). Subsequently, 500 μl of the Avicel slurry was added to the cell lysate, which was allowed to bind with rotation at 4°C for 1 h. The Avicel was pelleted by centrifugation at 20,000 × *g* for 2 min and washed three times each with 1 M NaCl/50 mM phosphate buffer (pH 7.5) and with 50 mM phosphate buffer (pH 7.5). The bound protein was eluted by heating at 105°C for 8 min in 1 × SDS sample buffer. The unbound and input fractions were concentrated by ultrafiltration (Microcon Ultracel YM-10, Millipore), mixed with 1/2 volume of 3 × SDS sample buffer, and heated as described above. The fusion protein in each fraction was detected by Western blot analysis, as described above.

## Results

### STATa-dependent expression of the *expL3* gene

**Figure 1:**
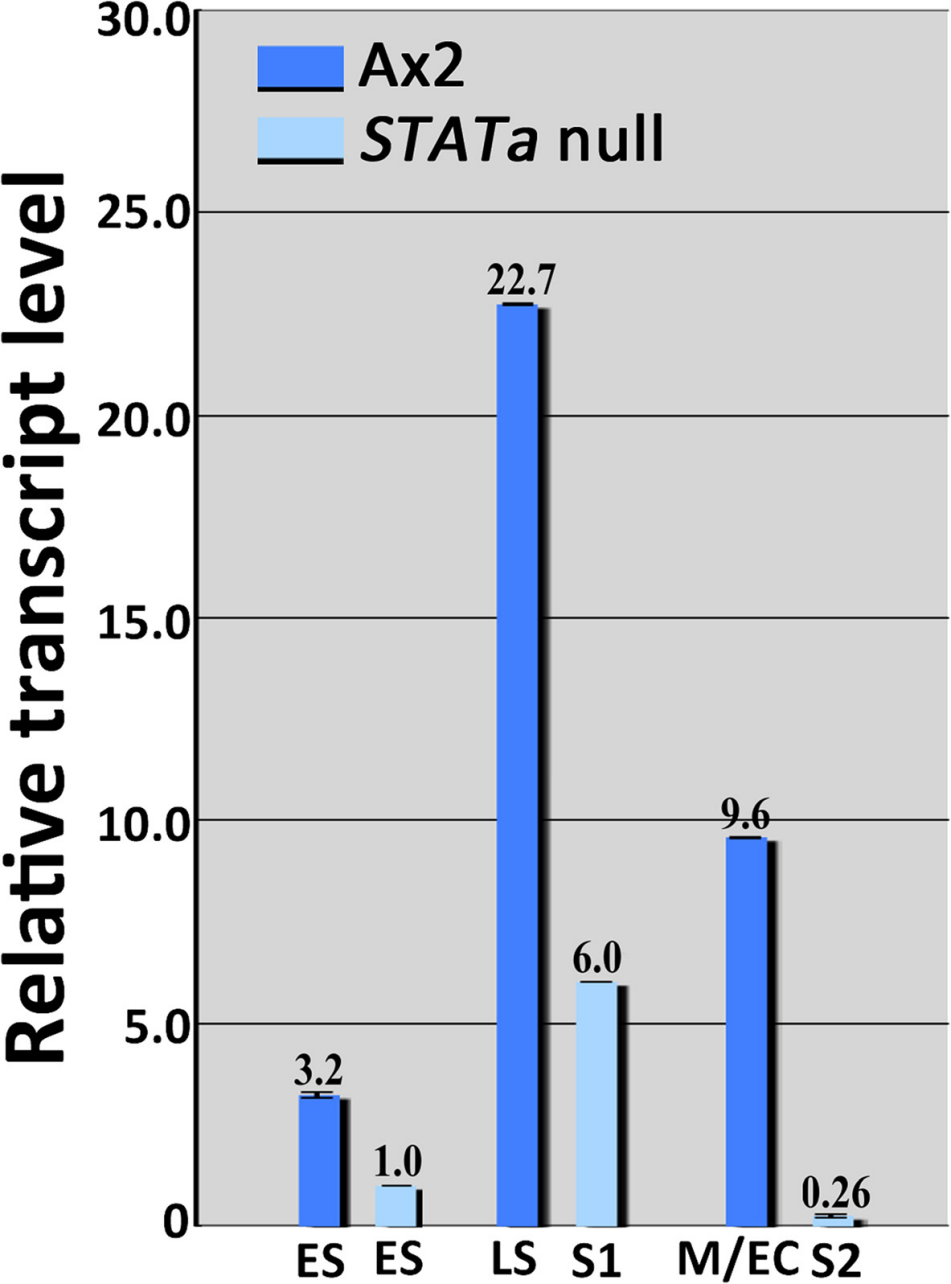
Comparison of *expL3* transcript levels in Ax2 and *STATa* null strains. Ax2 and *STATa* null cells were allowed to develop until the desired developmental stages. Total RNA was extracted from Ax2 cells (shown as blue bars) at the first finger/early slug (indicated as ES), late slug (LS), and Mexican hat/early culminant (M/EC) stages, and from *STATa* null mutant cells (shown as pale blue bars) at the first finger/early slug (ES), migrating slug after 20 h of development (S1), and slug at 24-h development (S2) stages, and used to amplify the specific *expL3* DNA fragment. The expression of *expL3* in Ax2 and *STATa* null cells was investigated by quantitative real-time PCR. The *IG7* transcript was used as a normalization control. The bar heights represent the average relative transcript level based on triplicate measurements, with the error bars. The expression of *expL3* in the *STATa* null early slug stage was set at 1.00. Statistically differences of expression level were analyzed by Student’s *t*-test, which showed all p-values were less than 0.01 between Ax2 and STATa null strain at comparable stages. To amplify the specific DNA fragment, we used 0.1 μg of total RNA and the primers: expL3-Fq and expL3-Rq (see Supplementary Figure 3a for their locations). The developmental stage is shown at the bottom of each column and the name of the strain is shown below each horizontal bar.

Previously, we showed that a *Dictyostelium* expansin-like gene, *expL7*, has crucial roles in morphogenesis during development (Ogasawara et al. 2009). Its expression is regulated by CudA (Wang and Williams 2010), where the expression of this gene in the tip-organizer cells is regulated by STATa (Fukuzawa and Williams 2000). Therefore, we examined the STATa-dependency of gene expression for other expansin-like family members. The majority of the genes examined in the family exhibited STATa-independent expression (see Additional file 2 and Additional file 3: Figures S1 for *expL4* and *expL6*), whereas *expL3* (dictyBase ID *DDB_G0276287*) exhibited STATa-dependent expression because according to quantitative RT-PCR, its transcript level was significantly reduced in the *STATa* null strain compared with that in the parental Ax2 strain at the corresponding stages (Figure 1).

### Expression of *expL3* is developmentally regulated

To elucidate the roles of the *expL3* gene, we investigated the similarity between *Dictyostelium* ExpL3 and ExpL7. The alignment obtained using CLUSTALW showed that the deduced amino acid sequence of ExpL3 shared 16% identity and 43% similarity with that of *Dictyostelium* ExpL7. Similarly, ExpL3 shared 17% identity and 59% similarity with *Arabidopsis thaliana* expansin, EXPA1. Similar to ExpL7, ExpL3 harbors the conserved motifs or domains that characterize the expansin found in *Arabidopsis* EXPA1, although the homology of some of the domains is weak, such as the numbers and locations of the conserved tryptophan residues in the cellulose-binding domain (see Additional file 2 and Additional file 3: Figure S2).

**Figure 2:**
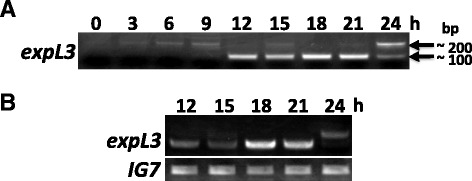
Temporal expression profiles of the *expL3* gene. **A**: Developmental time course of *expL3* splicing detected by semi-quantitative RT-PCR. Total RNA was extracted from Ax2 cells every 3 h and used as a template for the amplification of a specific *expL3* to detect DNA fragments with or without an intron. The image shows the RT-PCR results for *expL3* after 30 cycles of amplification. Note that after 30 cycles of amplification, the band intensity was almost saturated between 12–21 h. The band of approximately 200 bp contained an intron whereas the band of approximately 100 bp did not. The stages are loose aggregate (6 h), mound (9 h), tipped aggregate (12 h), slug (15 h), Mexican hat (18 h), culminant (21 h), and mature fruiting body (24 h), respectively. **B**: Expression profiles of the *expL3* gene during multicellular developmental stages. The upper row shows the RT-PCR results for *expL3* after 27 cycles of amplification before the PCR reaction saturated. *IG7* was detected using the same cDNA as a normalization control. The *IG7* DNA product (lower row) was detectable after 17 cycles of amplification.

To further characterize the *expL3* gene, we investigated the expression profiles of *expL3* both temporally and spatially, where we examined the relative *expL3* transcript level using semi-quantitative RT-PCR. The *expL3* gene has a single intron in the coding region; thus, PCR primers were designed for both sides of the intron (see Additional file 2 and Additional file 3: Figure S3A). The results showed that *expL3* expression is developmentally regulated, with a peak at approximately 18 h, which is the late slug to Mexican hat stage (Figure 2).

The *expL3* transcript was efficiently spliced only during the developmental stages between 12 h (tip stage) and 21 h (Mexican hat stage) because a smaller band that corresponded to the fragment without the intron was strongly amplified (Figure 2a). During the earlier stages until 9 h (mound stage), only a larger band that corresponded to the fragment with the intron was detected. Both bands were detectable at 24 h (fruiting body). These results indicate the posttranscriptional regulation of *expL3* in addition to transcriptional regulation, which is specific to the multicellular developmental stages.

### Expression of the *expL3* is prestalk-specific

**Figure 3:**
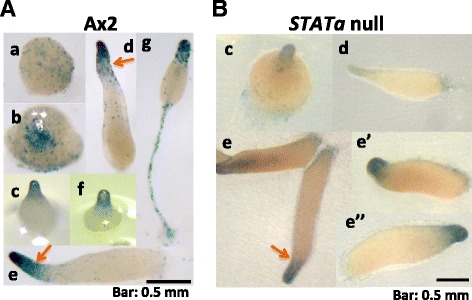
Spatial expression pattern of *expL3* promoter activity detected with a *lacZ* reporter construct. The *lacZ* reporter construct *expL3/lacZ* contained a 948-bp upstream promoter region fused to the β-galactosidase (*lacZ*) gene. Ax2 cells (panel **A**) and *STATa* null cells (panel **B**) transformed with *expL3/lacZ* were allowed to develop on filters until the mound (a), tip (b, c), first finger (d), slug (e), late slug (e’ and e” for *STATa* null), Mexican hat (f), and culminant (g) stages. To detect *expL3* expression, staining was carried out at 37°C for 4 h. Arrows indicate the position of pstO cells where the difference of staining pattern between Ax2 and *STATa* null strains were observed. The bars represent 0.5 mm.

To clarify the spatial expression pattern, we produced an *expL3/lacZ* reporter construct and the promoter activity was assayed in terms of its β-galactosidase activity. A scattered signal was detected at the mound stage (Figure. 3A-a). Furthermore, the signal was sorted to the prestalk (pst) region during tip formation (Figure 3A–b, c). During the first finger, slug, and Mexican hat stages, a strong signal was detected in the pstA region, whereas a weak scattered signal was detected in the pstO region (Figure 3A–d, e, f). Later, a signal was detectable in the stalk of the culminant (Figure 3A-g).

When the *STATa* null cells were transformed with the same construct, weaker staining of the pstA cells was visible during any of the multicellular stages, but almost no pstO or scattered staining was visible (Figure 3B). This indicates that STATa is necessary for adequate expression of the *expL3* gene, but it does not confer pstA-specificity.

### Overexpression of the *expL3* gene caused morphological aberrations

To examine the functions of the *expL3* gene, a mutant strain that lacked this gene was created via homologous recombination (Additional file 2 and Additional file 3: Figure S3). Three independent targeted clones (*expL3* null) were isolated, where the cells of these clones developed normally on non-nutrient agar plates and in other conditions. They formed normal looking fruiting body with calcofluor stained stalk and mature spores with normal shape and size (data not shown).

**Figure 4:**
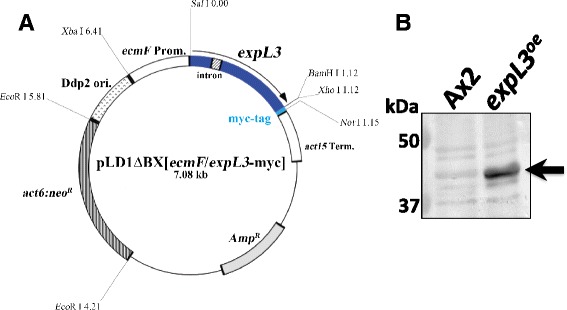
Overexpression of the ExpL3-myc protein driven by the pstA-specific *ecmF* promoter. **A**: Physical map of the overexpression construct of the ExpL3-myc fusion protein, pLD1ΔBX[*ecmF*/*expL3*-myc]. The late stage and pstA-specific promoter *ecmF* (Shimada et al. 2004) was used to drive the coding region of the *expL3* gene, which includes an intron. **B**: Expression of ExpL3-myc fusion protein detected by Western blot analysis. Total proteins were extracted from the slugs of the Ax2 strain (lane Ax2) and the ExpL3-myc overexpressing strain (lane *expL3* ^*oe*^), and then subjected to Western blot analysis, as described in the Materials and methods. The filter was probed with anti-c-Myc monoclonal antibody 9E10. The arrow indicates the position of the ExpL3-myc fusion protein (approximately 38 kDa).

Furthermore, we created strains that overexpressed the ExpL3-myc fusion protein using two different constructs. The first contained the *actin15* promoter to drive *expL3-myc* fusion gene, which was transformed into the Ax2 strain to yield Ax2/[*act15*]:*expL3*:*myc* (designated as *act15:expL3 myc*^*OE*^). When *act15:expL3 myc*^*OE*^ cells were allowed to develop on non-nutrient water agar plates, the development of the slug stage was delayed by 1–2 h, and they tended to migrate longer than Ax2 slugs (data not shown). The *actin15* promoter is not constitutive, and it peaks at 2–6 h before its transcription level gradually reduces as development proceeds (Cohen et al. 1986); therefore, the effect of its overexpression may not be retained during the later stages of development. To resolve this issue, we attempted to place the fusion gene under the control of its own *expL3* promoter. However, this construct was unstable and we could not create it successfully. Therefore, we attempted to use the *ecmF* promoter to replace the *actin15* promoter, and to create a new overexpression construct (Figure 4A) because the *ecmF* promoter activity is similar to that observed for *expL3* both temporally and spatially, i.e., late stages and pstA-specific (Shimada et al. 2004; also see Figures 2 and 3A). Overexpression of the fusion protein was confirmed in the resultant transformant of Ax2/[*ecmF*]:*expL3*:*myc* (designated as *expL3*^*oe*^) by Western blot analysis (Figures 4B and Additional file 2 and Additional file 3: Figure S4).

**Figure 5:**
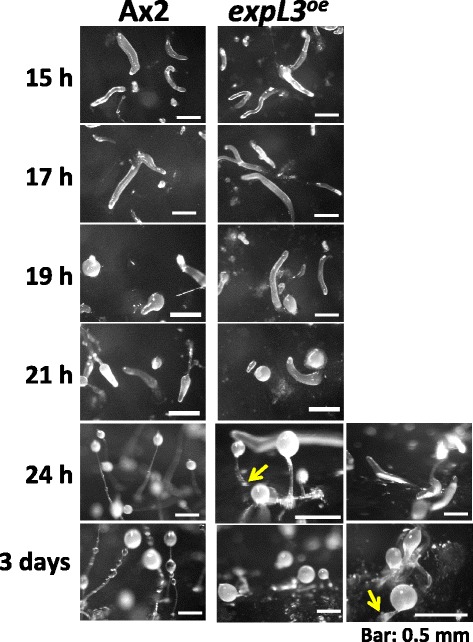
Delayed development and aberrant structure of the culminant caused by overexpression of ExpL3-myc protein. Cells of the parental Ax2 strain and ExpL3-myc overexpressing strain, where expression was driven by the *ecmF* promoter, Ax2/[*ecmF*]:*expL3*:*myc* (*expL3* ^*oe*^), were allowed to develop on non-nutrient water agar plates. The time of development is indicated at the left of each row and the strain is indicated above each column. After 24 h of development, although some (~40%) of the *expL3* ^*oe*^ strain formed culminant-like structures, majority (~60%) remained as slugs (the far right column). After 3 days of development, the structure formed was apparently abnormal because they formed a thick and short stalk, although they formed a spore head. Bars denote 0.5 mm. Arrows shows slightly unclear basal disc.

The *expL3*^*oe*^ cells developed normally on non-nutrient water agar plates until the first finger/early slug stage (approximately 15 h). However, the development of the *expL3*^*oe*^ started to delay at slug stage, the time when the ExpL3-myc protein became detectable (Additional file 2 and Additional file 3: Figure S4). The slugs tended to migrate for longer, where the majority (~60%) remained as slugs even after 24 h of development (Figure 5). A limited number of Mexican hat-like structures were observed at 21 h and culminant-like structures (culminants) were present among the slugs at 24 h. After 3 days of development, most of them (more than 95%) became culminants; however, they exhibited aberrant terminal structures, where a short (~1 mm long or shorter) but thick stalk-like structure supported the spore mass (Figure 5). They formed cellulose (Additional file 2 and Additional file 3: Figure S5), mature spore (data not shown) but they did not form an unequivocal basal disc. This indicates that the *expL3* gene causes morphological aberrations when it is overexpressed in the anterior pstA cells.

### ExpL3 protein binds to cellulose

**Figure 6:**
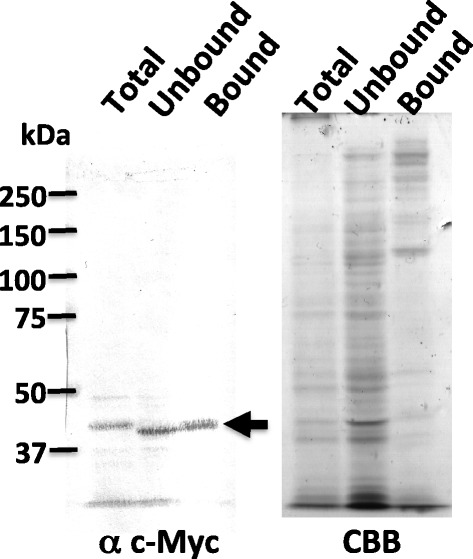
Binding of the ExpL3-myc fusion protein to microcrystalline cellulose (Avicel). The left panel shows the results of the Western blot analysis probed with the anti-c-Myc antibody 9E10. The right panel shows Coomassie brilliant blue R-250 (CBB) staining after SDS-PAGE using the same samples utilized in the Western analysis. The samples in each lane are: Total, input total cell lysate protein from the slug of *act15:expL3 myc* ^*OE*^ strain; Unbound, Avicel unbound flow-through fraction; and Bound, Avicel bound fraction. The arrow indicates the position of the ExpL3-myc fusion protein.

Most plant expansins localize in the cell wall and they are considered to disrupt noncovalent bonds among polysaccharides, as well as possessing a region with a homology to cellulose-binding domain; therefore, they are reported to bind cellulose (McQueen-Mason and Cosgrove 1995). We tested this hypothesis using *Dictyostelium* ExpL3, where we examined the binding activity of the myc-tagged ExpL3 using the *act15:expL3 myc*^*OE*^ strain (Figure 6). The cells were ground and allowed to bind to crystalline cellulose beads (Avicel). The ExpL3-myc protein was detected in the bound fraction (Figure 6, left panel), which indicates that ExpL3 is capable of binding to cellulose.

## Discussion

It has been reported that the *expL6* transcript is the only developmentally regulated member of this family of genes (Darley et al*.*^2003^), but we showed that the expression of the *expL3* gene is late stage-specific (Figure 2) in the present study. The reason for this discrepancy is unknown; however, the RT-PCR profile obtained in this study almost matched with the dictyExpress RNA-sequencing (RNA-Seq) database (Parikh et al., 2010; Additional file 1: Table S2). The RNA-seq database indicates that most of the expansin-like family genes in *Dictyostelium* are expressed in a stage-dependent manner (Additional file 1: Table S2). Based on the current results and our previous study of *expL7* (Ogasawara et al. 2009), we conclude that the expression levels of all expansin-like genes are developmentally regulated in *Dictyostelium*.

The *expL3* gene is expressed only in prestalk cells, i.e., strongly in pstA and weakly in pstO cells (Figure 3). Again, there was a discrepancy in the tissue specificity according to previous *in situ* hybridization results (Maruo et al. 2004; EST clone *SSI248*) and the β-galactosidase staining results obtained in the present study (Figure 3). This may have been caused by cross-hybridization of the probe used for *in situ* hybridization, although we cannot exclude the possibility that the promoter region upstream of the 5’ end point of the *expL3/lacZ* construct confers expression in prespore cells. The RNA-seq database indicates prestalk (pst) or prespore (psp) enrichment for expansin-like family member transcripts, i.e., *expL1*, *expL3*, *expL7*, and *expL9* transcripts are pst-enriched; *expL2* and *expL8* are psp-enriched; and *expL4*, *expL5*, and *expL6* exhibit no obvious tissue enrichment (Parikh et al., 2010; Additional file 1: Table S2). Thus, we conclude that the expression of the *expL3* gene is prestalk-specific.

The results of *expL3/lacZ* β-galactosidase staining in the *STATa* null mutant indicated that STATa may be involved in the strength of *expL3* expression but it does not contribute to pstA specificity, although STATa is activated in pstA cells. Alternatively, the *STATa* null mutant lacks most of the pstA cells, and thus only the weaker expression in pstO cells was detectable. STATa-dependent expression was investigated among the family of genes, except *expL9*, but no apparent STATa-dependency was observed other than that in *expL3* and *expL7*. These results suggest that the roles of these two expansin-like genes, i.e., *expL3* and *expL7*, are of particular importance during development.

The lack of the phenotype in the *expL3* null mutant might be an effect of the functional redundancy of the closely related genes, i.e., *expL1*–*9*, in the *Dictyostelium* genome. In contrast to the single null mutation, the overexpression of expansin or expansin-like genes in transgenic plants and *Dictyostelium* obtained the morphogenetic phenotype (Choi et al. 2003; Ogasawara et al. 2009). In agreement with these observations, overexpression of the ExpL3-myc protein via the pstA-specific *ecmF* promoter led to a developmental delay after slug formation and a morphological aberration during culmination (Figure 5). We do not know how the *Dictyostelium* ExpL3 protein exerts its effect on the morphology. However, it is possible that ExpL3 exerts its effect on cellulose in the stalk tube and that it regulates stalk elongation because we found that ExpL3 binds cellulose (Figure 6). We did not test whether the ExpL3 protein has a cell wall-loosening activity. If it possesses this activity, overexpression of the ExpL3 protein in stalk cells might weaken the stalk strength against gravity to yield the short, broad stalk.

Actually, *expL3* and *expL7* do not share many some features except STATa-dependency. The *expL7* is reported CudA-dependent (Wang and Williams, 2010), but we have preliminary result *expL3* is different (data not shown). Cell type-specificity is also different; pstA-specific for *expL3* (Figure 3) and tip-organizer cell-specific for *cudA* (Fukuzawa and Williams, 2000). Therefore, we think these two genes may behave independently. Indeed, double overexpressor strain of *expL3* and *expL7* genes (*expL3*^*OE*^/*expL7*^*OE*^) displayed the phenotype as if it was like that seen in the *expL3*^*OE*^ until culmination (Figure 5), after that it was like that seen in the *expL7*^*OE*^ (Ogasawara et al. 2009) (data not shown). Whatever the case, the phenotype of *expL3*^*oe*^ implies the involvement of ExpL3 in morphogenesis during slug migration and culmination in *Dictyostelium*.

## Additional files

Additional file 1: Table S1. Sequence of primers used in the PCR reactions. **Table S2.** Summary of the expression profiles of the expansin-like family genes in *Dictyostelium*. Additional file 2:
**Methods and Figure legends for Additional file** 3. Additional file 3: Figure S1. Developmental time course of *expL4* and *expL6* gene expression determined by semi-quantitative RT-PCR. **Figure S2.** Amino acid sequence of ExpL3 and similarity of the domain organization to plant expansin. **Figure S3.** Creation of the *expL3* null mutant. **Figure S4.** Stage-specific expression of ExpL3-myc via the *ecmF* promoter in the overexpressing strain. **Figure S5.** Conformation of ExpL3-myc overexpression in pstA cells in the overexpressing strain.
