# Supplementary material for: Implications of expansin-like 3 gene in Dictyostelium morphogenesis
Source: Springerplus. 2015 Apr 19;4:190. doi: 10.1186/s40064-015-0964-0 (PMC4408306; doi:10.1186/s40064-015-0964-0)
Supplement: Additional file 1: Table S1. — Sequence of primers used in the PCR reactions. Table S2. Summary of the expression profiles of the expansin-like family genes in Dictyostelium. [file 40064_2015_964_MOESM1_ESM.pdf]

Table S1 Sequence of primers used in the PCR reactions

Primer Name	Sequence		Experiment
IG7-Fq	5'-GCACCTCGATGTCGGCTTAA-3'		quantitative PCR
IG7-Rq	CACCCCAACCCTTGGAAACT		quantitative PCR
IG7-P	FAM-CATCCCGGAGTTGAAA-MGB	(TaqMan)	quantitative PCR
expL-3-Fq	GGTCCATTGGGTCCTGGTAA		quantitative PCR
expL-3-Rq	ACCACATTGAGCACCATTTTGA		quantitative PCR
expL3-G1	GGTCGTAATGTTGATAAAGATTTATCTG		cloning, KO screening
expL3-G2	GGTGCCCAGCCCAGTTACAACCATGAACC		cloning, KO screening
Hyg-1	GACAGACGTCGCGGTGAGTTCAGG		KO screening
expL3-KO	GATGGACATATATCAATCTTGTTAATCC		KO screening
expL3-G4-Sal	ACGCGTCGACATGAAATTCAATACTATCTTTTTGG		vector construction
expL3-G5-Bam	CGGGATCCATAACAAGAAATTTGGTAAAAG		vector construction
expL3-G0-Xba	GCTCTAGACCTCCACCAGGTACTTTAATC		*lacZ* construct
expL3-G3-Bgl	GAAGATCTCAAAAAGATAGTATTGAATTTC		*lacZ* construct
expL4-F	GGTCCATTGGGTCCTGGTAA		semi-quantitative PCR
expL4-R	ACCACATTGAGCACCATTTTGA		semi-quantitative PCR
expL6-F	CATGCATCATGTGGTTTCGAA		semi-quantitative PCR
expL6-R	AATGCAACAACCAAACGATTACC		semi-quantitative PCR
expL3-RT-1	CTGATTCATGTCATGATGCAGGTTATTGTC		semi-quantitative PCR
expL3-RT-2	GAGTATTTGAATTTTGATTGTTTGAAG		semi-quantitative PCR
expL3-G7-i	TCCTGGTAACTATATGATTGCAGCTCTTGG		semi-quantitative PCR
expL3-G8-i	CTGTAACTGAAGCATTTTTAGAATTTG		semi-quantitative PCR

The name of each primer is shown in the left column. Its sequence is shown in the right column in 5' to 3' direction. FAM, 6-FAM (6-carboxyfluorescein); MGB, Minor Groove Binder.

Table S2 Summary of the expression profiles of the expansin-like family genes in *Dictyostelium*

Gene	dictyBase Gene ID	GenBank Accession Number	RNA-Seq database		
			Expression peak	$\log_2$(psp/pst)	Prespore or prestalk enrichment
expL1	DDB_G0267846	EAL73376	0 h then gradually decreases	−3.341	pst
expL2	DDB_G0284677	EAL65108	24 h, no expression until 20 h	2.659	psp
expL3	DDB_G0276287	EAL69290	12–16 h	−4.0135	pst
expL4	DDB_G0279847	EAL67537	0 h* and 12 h	0	no obvious bias
expL5	DDB_G0276937	EAL68970	4 h and 16 h	0.067	no obvious bias
expL6	DDB_G0267844	EAL73375	8 h* and 20 h and relatively high level in late stages	−0.295	no obvious bias
expL7	DDB_G0288331	EAL63262	20–24 h	−2.024	pst
expL8	DDB_G0288331	EAL60943	8–20 h	1.123	psp
expL9	DDB_G0293148	EAL60926	16 h	−2.934	pst

Data were obtained from the RNA-sequence database for each gene in dictyBase (http://dictybase.org/).

* Uncertain peak because the profile was discontinuous or unusually protuberant
