# Supplementary material for: Implications of expansin-like 3 gene in Dictyostelium morphogenesis
Source: Springerplus. 2015 Apr 19;4:190. doi: 10.1186/s40064-015-0964-0 (PMC4408306; doi:10.1186/s40064-015-0964-0)
Supplement: Additional file 2: — Methods and Figure legends for Additional file 3. [file 40064_2015_964_MOESM2_ESM.pdf]

Additional file 2

Methods

***expL3* knockout construct and screening of the *expL3* null strains**

The genomic fragment that corresponded to a part of the promoter and the full-length ORF region was amplified by PCR using the oligonucleotides expL3-G1 and expL3-G2 as primers, and genomic DNA from the Ax2 strain as the template, where the amplified fragment was subcloned into pCR2.1-TOPO (Invitrogen) to yield pTOPO[*expL3*(G1/G2)]. A unique *Bam*HI site in the pTOPO[*expL3*(G1/G2)] vector was destroyed by fill-in reaction after digestion with *Bam*HI to obtain pTOPO[*expL3*(G1/G2)ΔBamHI]. The resultant vector was digested with *Nde*I in the ORF region (Additional file 3: Figure S3) and the site was converted into a *Bam*HI site by ligation with *Bam*HI-linker, thereby introducing a unique *Bam*HI site into the ORF region to create pTOPO[*expL3*(G1/G2)+*Bam*HI]. The vector was digested and the *Bam*HI cassette of the hygromycin B resistance gene (Hyg^R) was introduced to obtain a knockout construct pTOPO[*expL3*KO:Hyg^R] (Additional file 3: Figure S3).

The target vector was digested with *Not*I and *Spe*I. After phenol/chloroform extraction of the vector DNA, Ax2 cells were transformed by electroporation. The transformants were selected in 96-well plates containing HL5 medium supplemented with 36 μg/ml hygromycin B. To screen for the clones where the *expL3* gene was successfully targeted, genomic DNA from each clone was amplified by PCR using two pairs of primers: expL3-G1 and expL3-G2, and Hyg-1 and expL3-KO (see Additional file 3: Figure S3).

Microscopic analysis of Myc fusion protein

To detect ExpL3-myc, multicellular structures at the late slug or culminant (24 h development) stages were allowed to settle on Teflon-coated slides and fixed with cooled methanol at −25 °C for 30 min (Shimada et al. 2010), followed by successive washes with 70% methanol/PBS, 35% methanol/PBS, and PBS for 5 min each. The fixed samples were incubated with anti-c-Myc monoclonal antibody 9E10 (1:200 dilution; Wako) as a primary antibody for >20 h at 4 °C. Next, they were washed in PBS and incubated with Alexa Fluor 488-conjugated goat anti-mouse IgG antibody (1:200 dilution; Invitrogen) as a secondary antibody for >20 h at 4 °C. Finally, the samples were mounted with ProLong Gold antifade reagent (Invitrogen) and visualized with an Olympus epifluorescence microscope (model IX71; Olympus, Japan). Microscopic images were captured as described previously (Kunii et al. 2014).

Figure legends

Figure S1 Developmental time course of *expL4* and *expL6* gene expression determined by semi-quantitative RT-PCR. Total RNA was extracted from Ax2 cells (upper panel) at the vegetative growth (0 h), before aggregation (3 h), late aggregation (6 h), mound (9 h), tipped aggregate (12 h), slug (15 h), Mexican hat (18 h), culminant (21 h), and fruiting body (24 h) stages, and from the *STATa* null mutant cells (lower panel) at the mound (12 h), tipped aggregate (16 h), and slug (20, 24, 30 h) stages, and used to amplify the specific *expL4* (upper row) and *expL6* (middle row) DNA fragments, as described in the Materials and methods. Each DNA was detectable after 30 cycles of amplification before the PCR reaction saturated. Ribosomal RNAs (1 μg total RNA/lane) were electrophoresed and stained with ethidium bromide as the loading standard (data not shown). *IG7* was detected using the same cDNA as a normalization control. The *IG7* DNA product (lower row)

was detectable after 17 cycles of amplification. Note that the image of the *IG7* DNA is the same as that used in Figure 2 because these reactions were performed at the same time as the semi-quantitative RT-PCR of *expL3* (Figure 2B).

Figure S2 Amino acid sequence of ExpL3 and similarity of the domain organization to plant expansin. A: Schematic representation of the conserved motifs in plant α-expansin, EXPA1. The positions of the conserved motifs are not shown to scale. The consensus sequences or residues are shown below or in the boxes. Box colors correspond to the colors in the alignment in **C**. The illustration was modified from Li et al. (2002) and adapted from Ogasawara et al. (2009). The N-terminal domain (Domain 1) shared weak homology with the catalytic domain of the glycoside hydrolase family 45 (GH45) proteins. The C-terminal domain (Domain 2) shares similarity with a type-A cellulose-binding domain (Li et al. 2002; Sampedro and Cosgrove 2005; Nikolaidis et al. 2014). **B**: Alignment of the amino acid sequences of *Dictyostelium* ExpL3 and ExpL7 (Ogasawara et al. 2009). The sequences were aligned using the CLUSTALW version 1.83 with the default settings (http://clustalw.ddbj.nig.ac.jp/index.php?lang=ja). Amino acid residues that are identical in the two proteins are shown by asterisks and those representing conservative changes are indicated by dots. **C:** Alignment of the amino acid sequences of ExpL3 and *Arabidopsis thaliana* EXPA1 (GenBank accession number AEE34945). The sequences were aligned as described above.

Figure S3 Creation of the *expL3* null mutant. A: Schematic representation of the knockout construct used for targeted mutagenesis of the *expL3* gene. The boxed region was subcloned into the vector and the long arrows in the blue and pink boxes indicate the orientation of *expL3* and Hyg^R cassette, respectively. The gray region in the box represents an intron. The solid bars that represent the flanking regions of *expL3* were not included in the knockout construct. The names of the primers

and the orientations used for screening the null mutant are shown as short arrows in the respective positions. The primers used in Figures 1 and 2 are also indicated. The Hyg^R cassette is not shown to scale. **B**: Screening of the *expL3* null mutant. PCR was carried out using genomic DNA extracted from Ax2 or the *expL3* null mutant (KO clone #1, #7, #16) as templates and with the primer pairs: expL3-G1⁄expL3-G2 (upper panel) and Hyg-1⁄expL3-KO (lower panel). The arrows in the upper panel indicates a fragment containing the Hyg^R cassette (3.7 kbp), which was amplified from the mutant cells, and a fragment without the Hyg^R cassette (2.3 kbp), which was amplified from the Ax2 cells. The arrow in the lower panel denotes a fragment (1.2 kbp) amplified only from the targeted cells. The arrowhead shows the artificially amplified fragments. **C**: Confirmation of gene targeting based on semi-quantitative RT-PCR with total RNA as the templates. Total RNA was extracted from late slugs of the Ax2 strain (lane Ax2) and the null strains (lanes KO #1, #7, #16). After synthesis of cDNAs from 0.25 μg total RNA, 27 cycles of PCR were performed with the primer pair: RT-1 and RT-2. The sequences of the primers are listed in Supplementary Table 1. The arrow represents a fragment without the Hyg^R cassette (0.1 kbp), which was amplified from the Ax2 cells. The arrowhead represents a fragment containing the Hyg^R cassette (1.5 kbp), which was amplified from a putative read-through transcript in the mutant cells because no band was detected after PCR in the same conditions without adding reverse transcriptase (data not shown).

Figure S4 Stage-specific expression of ExpL3-myc via the *ecmF* promoter in the overexpressing strain. Cells of Ax2/[*ecmF*]:*expL3*:*myc* (*expL3*^{oe} strain) were allowed to develop until the desired stage on non-nutrient water agar plates and total protein samples were prepared and solubilized, as described in the Materials and methods. The upper panel shows the Western blot analysis probed with anti-c-Myc monoclonal antibody 9E10, as described in the legend of Figure 4B. The time of development for the sample is indicated at the top of each lane. Lane M denotes the protein size markers. The lower panel

shows the gel stained with Coomassie brilliant blue R-250 (CBB). Approximately 10–15 μg of total protein was loaded in each lane, except for the 4 h sample where slightly more protein was loaded. The expression of the ExpL3-myc protein peaked at 24 h when the developmental stage of the *expL3*^{oe} strain mostly comprised the slug (see Figure 5).

Figure S5 Conformation of ExpL3-myc overexpression in pstA cells in the overexpressing strain. Cells of Ax2/[*ecmF*]:*expL3*:*myc* (*expL3*^{oe} strain) were allowed to develop for 24 h until the slug stage on non-nutrient water agar plates, before they were fixed and detected as described above. **A**: The pstA cell localization of ExpL3-myc. In addition to the strong signal in pstA cells, scattered ExpL3-myc signals were also visible in pstO, the prespore region, and rear guard cells. **B**: Production of cellulose in the stalk-like structure of the *expL3*^{oe} strain. (a) Bright field image. (b) ExpL3-myc localization as detected above. (c) After immunohistochemical staining, the culminant was stained with 0.1% (w/v) Calcofluor in the dark at room temperature for 5 min to visualize cellulose. (d) Merged image of (b) and (c). Bars denote 0.5 mm.

References

Kunii M, Yasuno M, Shindo Y, Kawata T (2014) A *Dictyostelium* cellobiohydrolase orthologue that affects developmental timing. Dev Genes Evol 224:25–35.

Li Y, Darley CP, Ongaro V, Fleming A, Schipper O, Baldauf SL, Mcqueen-Mason SJ (2002) Plant expansins are a complex multigene family with an ancient evolutionary origin. Plant Physiol 128:854–864.

Nikolaidis N, Doran N, Cosgrove DJ (2014) Plant expansins in bacteria and fungi: evolution by horizontal gene transfer and independent domain fusion. Mol Biol Evol 31:376–386.

Ogasawara S, Shimada N, Kawata T (2009) Role of an expansin-like molecule in *Dictyostelium* morphogenesis and regulation of its gene expression by the signal transducer and activator of transcription protein Dd-STATa. Develop Growth Diff 51:109–122.

Sampedro J, Cosgrove DJ (2005) The expansin superfamily. Genome Biol 6:242.

Shimada N, Inouye K, Sawai S, Kawata T (2010) SunB, a novel SUN domain-containing protein required for development of *Dictyostelium discoideum.* Develop Growth Diff 52:577–590.
