# Supplementary material for: Implications of expansin-like 3 gene in Dictyostelium morphogenesis
Source: Springerplus. 2015 Apr 19;4:190. doi: 10.1186/s40064-015-0964-0 (PMC4408306; doi:10.1186/s40064-015-0964-0)
Supplement: Additional file 3: Figure S1. — Developmental time course of expL4 and expL6 gene expression determined by semi-quantitative RT-PCR. Figure S2. Amino acid sequence of ExpL3 and similarity of the domain organization to plant expansin. Figure S3. Creation of the expL3 null mutant. Figure S4. Stage-specific expression of ExpL3-myc via the ecmF promoter in the overexpressing strain. Figure S5. Conformation of ExpL3-myc overexpression in pstA cells in the overexpressing strain. [file 40064_2015_964_MOESM3_ESM.pdf]

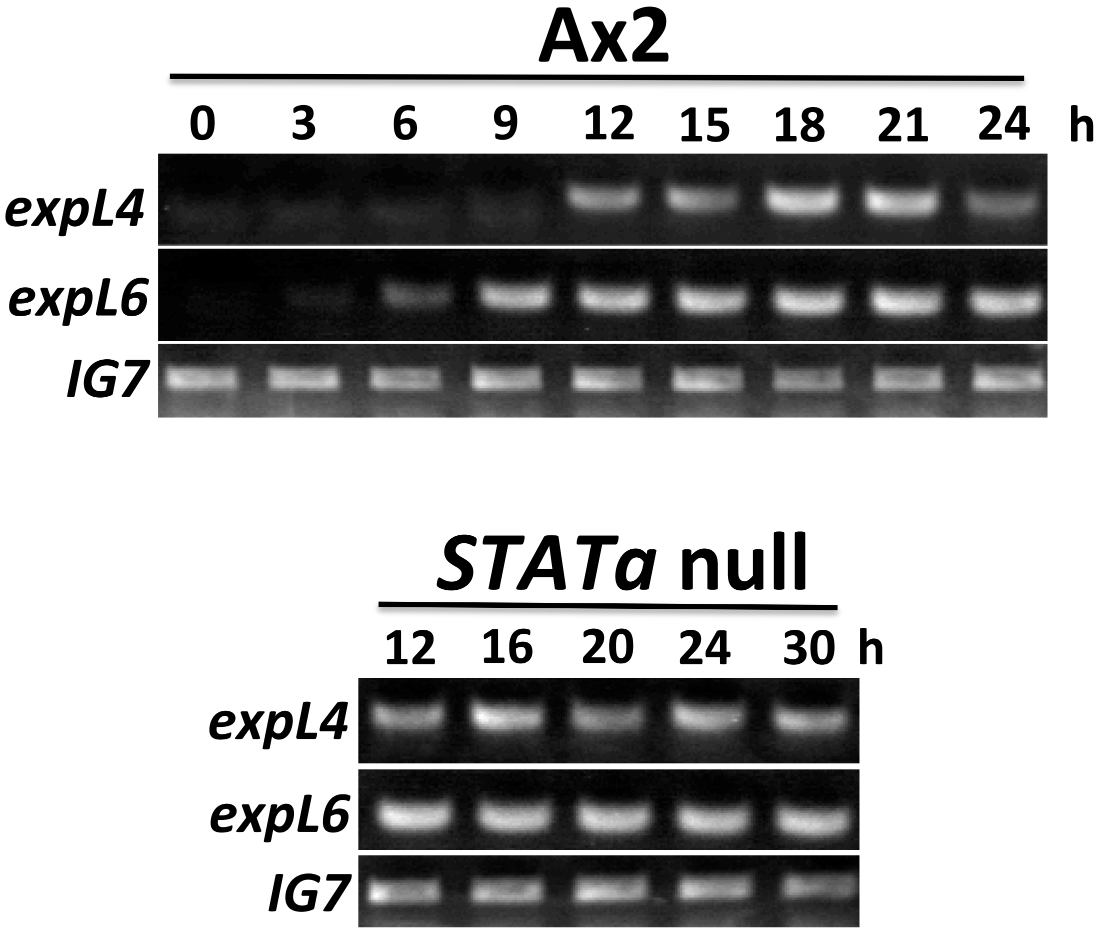
Ax2
0 3 6 9 12 15 18 21 24 h
expL4
expL6
IG7
STATa null
12 16 20 24 30 h
expL4
expL6
IG7

Figure S1

A

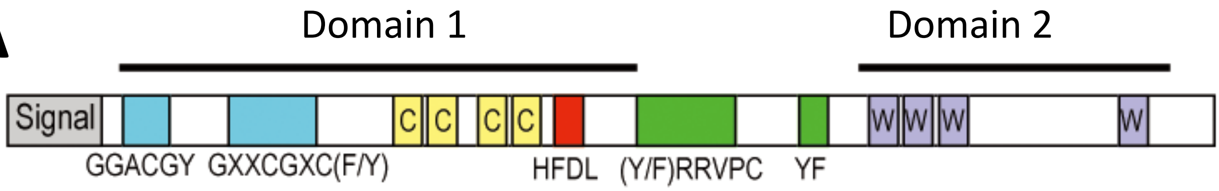

B

```
ExpL3        1   MKFNTIFLVLSIVKFILISAQSCPFSQSIINGASATFYTAIDAGNCGFEKLNGPLGPGNY
ExpL7        1   -MRLLGSLILTLSLIASAFSSSVPLTLCMS-GRAQGTESLNKSGSCEYGAYNGPTGPG-T
                        *:*::  :    :.* *:: .:  * :    :  .:*.* :    *** ***

ExpL3       60   MIAALGSKLYQNGAQCGQCFKISNSKNASVTVMATDSCHDAGYCQRDNHFDLSPAAFSIL
ExpL7       57   LTATLNEYFYSSGVKCGDCFEVSGPK-GKTVVRVVNFCSAGTCPSERPLFMLTPDAFQEI
                 : *:*.. :*..*.:**:**::*..* ....* ..: *   .    ..    * *:* **. :

ExpL3      120   GPQSQGVLDGLSYVKVPCEVSGNVKIMMKDGSNDFWTSFFVFNSKVIIKQVSIKLSNSNQ
ExpL7      116   SSDPLSVVYDAGFRKVSCDASGPIKAQVSEDSSKYYVKLLIFNNEVGIQSVTIKGKDMSA
                 ..:. .*: . .: **.*:.** :*  :.:.*..::.::**.:* *:.*:** .: .

ExpL3      180   FVPLSQTTYNYWPTSITGGQFHVRIESIGGEFIYVTIPKVE---SRKVYETSGQFSTSCS
ExpL7      176   PVTMVRQGSAQFVWSQAGKEMMFPATVVSSQYGGSVTMTMNSLSMDILKFSGNFVAPKS
                  *.: :      :  * :* :: .    : ..    ::. .    *  .: :  **:* :. *

ExpL3      237   NLNENNPIN---------------------------------------------------
ExpL7      236   SIIKNAPASCSLSASPLAIYQNGLTEGWNYWSSRSYSQINTTDSSSHSVGSTKSLSLVLM
                 .: :* *  .

ExpL3      246   ------------YKPQTFNSQQTSNNQNSNTQTPTKQPSPNSQN-------FIPSYCQQY
ExpL7      296   GSSSALTLARSGDFETTYFTGIKFNMKANTTMSGLRVYFPNEQNKYWTPSSPITTSWATY
                             *: :   . * : ..* :  :    **.**         *.:     *

ExpL3      287   IQKPNYIFAKESKEMLVLNENENIESNSLKLLPNFLLLILIILLNINF------------
ExpL7      356   TVPFTSLQHKTIESAFTFANTENINVHINIDNIHFIASPSSVTTGYANGNETANSGLATT
                     . :   *  :. :.: :.***: :       :*:      :   .

ExpL3            ------------------------------------------------------------
ExpL7      416   TVASGGSGASGVATSSHTGVSSSSSTASSTASSTASSIASSTASSSASSTSVSSTTAGGK

ExpL3            ------------------------------------------------------------
ExpL7      476   TTSGGSGISTSGITGSGDSMAASTSKTTSNPTGKTTGMTGSSIDHSESHSSDEHHSSSSI

ExpL3            -----------------
ExpL7      536   IKASLLLVSAALAFASL
```

C

```
EXPA1   ---MALVTFLFIATLGAMTSHVNGYAGGGWVNAHATFYGGGDASGTMGGACGYGNLYS-Q 56
ExpL3   MKFNTIFLVLSIVKFILISAQSCPFSQSIINGASATFYTAID-----AGNCGFEKLNGPL 55
            ::. .* *..:  ::::   :: .   .* **** . **.   .*:**:   * .

EXPA1   GYG-TNTAALSTALFNNGLSCGACFEIRCQNDGKWCLPGSIVVTATNFCPPNNALPNNAG 115
ExpL3   GPGNYMIAALGSKLYQNGAQCGQCFKISNSKNASVTVMATDSCHDAGYCQRDNHFDLSPA 115
        * *    ***.: *::** .** **:*  .::..  : .:      :.:*  :* :   ...

EXPA1   GWCNP-PQQHFDLSQPVFQRIAQYRAGIVPVAYR--------------------RVPCVR 154
ExpL3   AFSILGPQSQGVLDGLSYVKVPCEVSGNVKIMMKDGSNDFWTSFFVFNSKVIIKQVSIKL 175
        .:.    **.:   *.    : ::.    :* * :   :                :*.

EXPA1   RGGIRFTINGHSYFNLVLITNVGGAGDVHSAMVKGS-------RTGWQAMSRNWGQNWQS 207
ExpL3   SNSNQFVPLSQTTYNYWPTSITGGQFHVRIESIGGEFIYVTIPKVESRKVYETSGQFSTS 235
         .. :*.  .:: :*     : .**  .*:    : *.         :.  : : .. **    *

EXPA1   NSYLN-GQSLSFKVTTSDGQTIVSNNVANAGWSFGQTFTGAQLR---------------- 250
ExpL3   CSNLNENNPINYKPQTFNSQQTSNNQNSNTQTPTKQPSPNSQNFIPSYCQQYIQKPNYIF 295
         * ** .:.:.:*  * :.*   .*: :*:  .   *. ..:*

EXPA1   -----------------------------------------
ExpL3   AKESKEMLVLNENENIESNSLKLLPNFLLLILIILLNINF 335
```

Figure S2

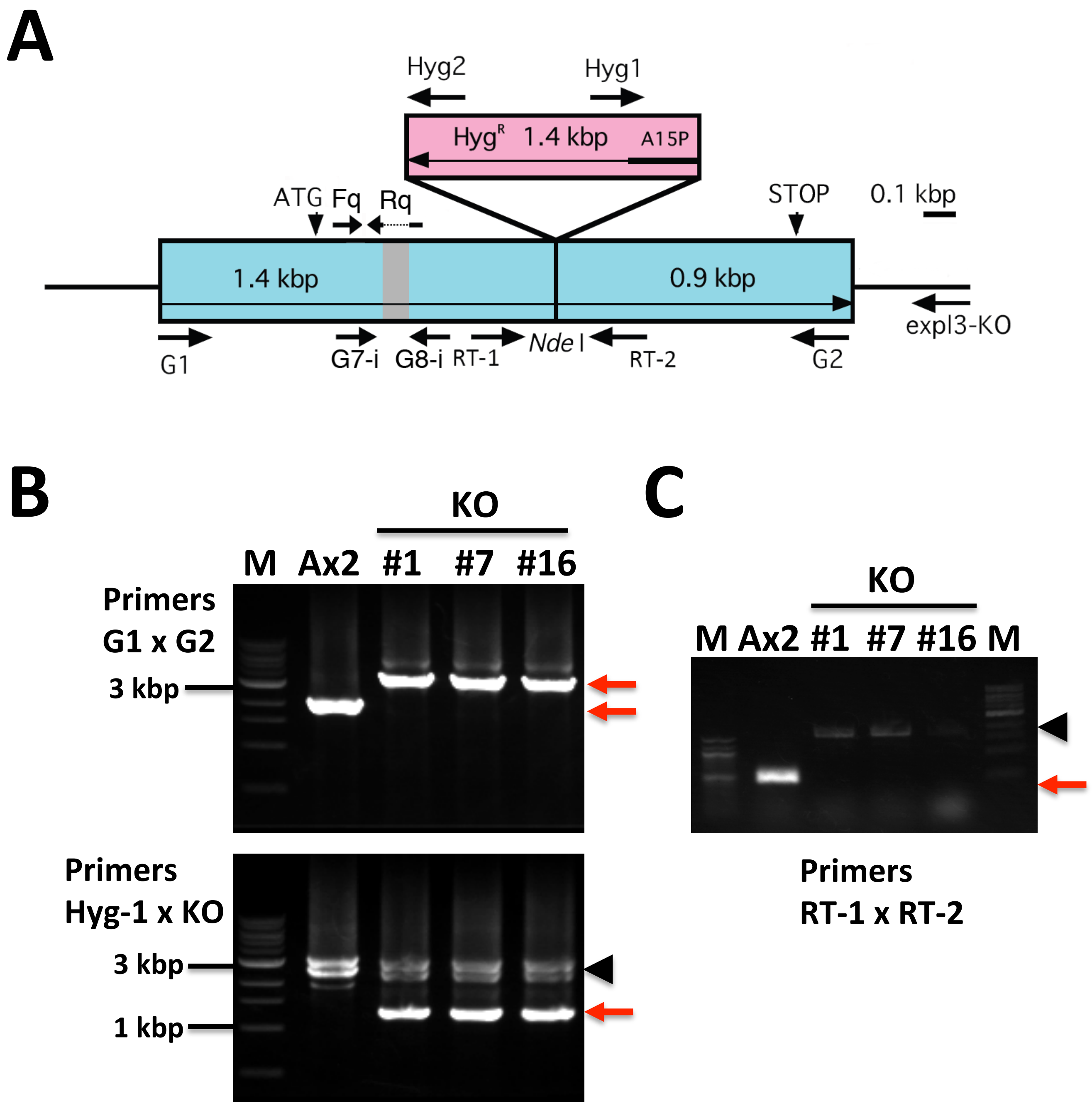
A
Hyg2
Hyg1
HygR 1.4 kbp
A15P
ATG
Fq
Rq
STOP
0.1 kbp
1.4 kbp
0.9 kbp
expl3-KO
G1
G7-i
G8-i
RT-1
Nde I
RT-2
G2
B
KO
M Ax2 #1 #7 #16
Primers
G1 x G2
3 kbp
Primers
Hyg-1 x KO
3 kbp
1 kbp
C
KO
M Ax2 #1 #7 #16 M
Primers
RT-1 x RT-2

Figure S3

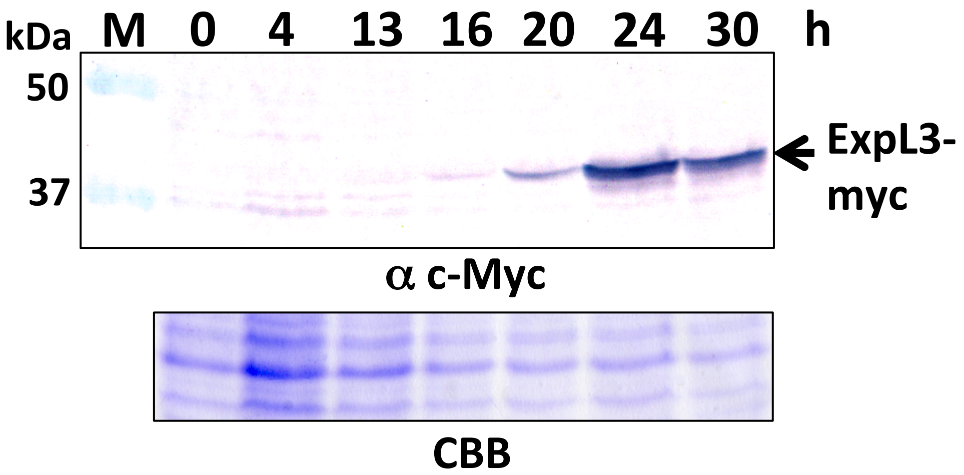
kDa
M
0
4
13
16
20
24
30
h
50
37
ExpL3-myc
α c-Myc
CBB

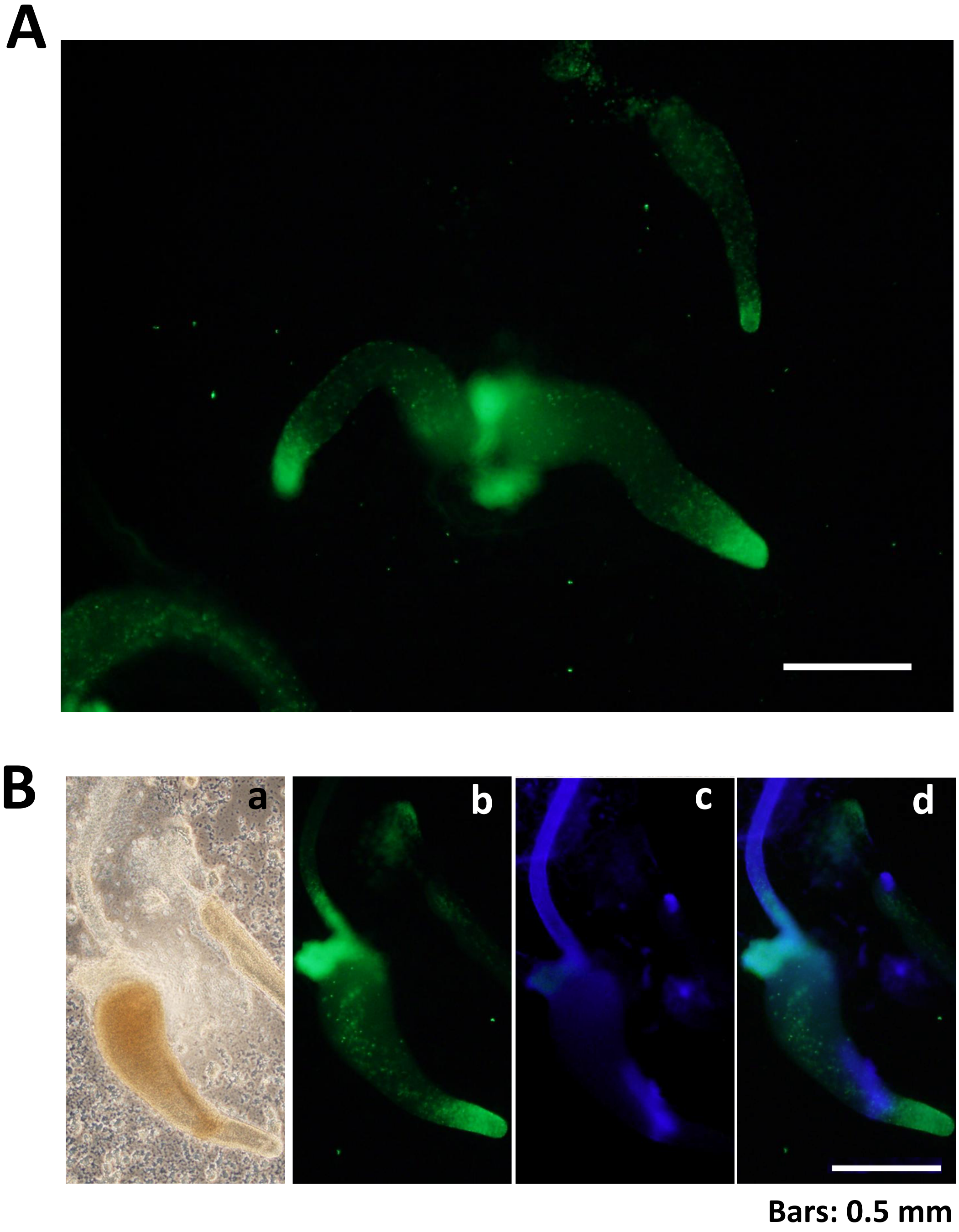

Figure S5
